# Investigation of the correlation between AGRN expression and perineural invasion in colon cancer

**DOI:** 10.3389/fmolb.2024.1510478

**Published:** 2024-12-03

**Authors:** Lei Chen, Haijia Zhang, Kaiyue Gao, Fanqi Meng, Funing Yang, Jiannan Li, Lijie Wang, Jiandong Tai

**Affiliations:** ^1^ Department of Colorectal and Anal Surgery, General Surgery Center, The First Hospital of Jilin University, Changchun, China; ^2^ Pediatric Outpatient Clinic, The First Hospital of Jilin University, Changchun, China; ^3^ Department of General Surgery, The Second Hospital of Jilin University, Changchun, China

**Keywords:** colon cancer, perineural invasion, AGRN, bioinformatics, immunohistochemistry

## Abstract

**Background and Purpose:**

Colon cancer is one of the most common gastrointestinal malignancies. According to the traditional view, the primary modes of transmission include direct dissemination, hematogenous metastasis, and lymph node metastasis. In recent years, the role of perineural invasion (PNI) in the spread and metastasis of tumors has received immense attention. However, there are still relatively few reports on the potential mechanisms and biomarkers of PNI occurrence and development in colon cancer.

**Method:**

We identified genes linked to the onset and progression of PNI in colon cancer using bioinformatics tools and extensive databases. Gene function enrichment analysis was used to explore the potential roles of these genes in tumor proliferation, invasion, and PNI. A collection of postoperative pathological specimens from colon cancer patients who underwent surgery, related clinicopathological data, and immunohistochemistry were used to validate AGRN expression in PNI tissues.

**Results:**

Bioinformatics analysis revealed that AGRN is overexpressed in colon cancer tissues and correlates with poor patient prognosis. The findings from gene association and enrichment studies indicate that AGRN and its associated genes may play a role in PNI development and progression in colon cancer by simultaneously enhancing tumor cell invasion and neural cell growth. Immunohistochemical analysis of clinical samples confirmed that AGRN expression is elevated in colon cancer tissues with PNI.

**Conclusion:**

We found that AGRN is significantly overexpressed in colon cancer tissues exhibiting PNI and is linked to poor patient survival. AGRN and its related genes may contribute to PNI by promoting tumor cell invasion and neural cell growth. Hence, AGRN may play a crucial role in the initiation and progression of PNI in colon cancer.

## 1 Introduction

Colon cancer is the most prevalent malignant tumors (Dekker et al., 2019). The available treatment options include endoscopic techniques, surgical excision, preoperative radiotherapy, systemic therapies, local ablative methods for metastases, and palliative approaches such as chemotherapy, targeted therapy, and immunotherapy (Ghidini et al., 2018). For patients without distant metastasis and who have surgical opportunities, surgical resection is a crucial treatment modality (Biller and Schrag, 2021). However, a portion of patients still experience recurrence and metastasis after surgical resection. Therefore, actively exploring the relevant molecular mechanisms of tumor metastasis pathways is of utmost importance (Ciardiello et al., 2022). Perineural invasion (PNI) may act as an additional metastatic pathway for colon cancer, closely related to severe cancer pain in advanced cases. It is identified when tumor cells encircle more than 33% of the nerve’s circumference and invade any of the three layers of the neural structure (Wang H. et al., 2023).

The mechanism of PNI may involve multiple interrelated steps between nerves and tumors, including nerve proliferation, tumor cell invasion, and the peritumoral microenvironment (Reavis et al., 2020; Hutchings et al., 2020). Due to the complexity of its mechanisms, the advantages of bioinformatics for large-scale gene screening are increasingly valued by researchers in exploring PNI progression (Guo J. A. et al., 2021; Huang et al., 2018; Schmitd et al., 2022). It was reported that neurotrophic factors, chemokines, and neurotransmitters are important molecular mediators that promote the development of PNI (Liebig et al., 2009). Neurotrophic factors, including the nerve growth factor (NGF) family, glial cell-derived neurotrophic factors and their receptors, the midkine (MDK) family, and axon guidance genes, play key regulatory roles in PNI (Yao et al., 2014; Jurcak et al., 2019). In addition, certain chemokines, such as CX3CL1, CXCR4, and CXCL12, expressed by neurons, are closely related to PNI formation. Studies have shown that blocking the CXCL12-CXCR4 signaling pathway significantly inhibits tumor growth, reduces nerve damage, and decreases PNI levels in tumor tissue (Xu et al., 2015). Moreover, the neurotransmitter catecholamine, through interaction with ADRB2, promotes NGF secretion and increases neural density in tumor tissues, thereby accelerating PNI onset and spread (Renz et al., 2018). This suggests that neurotransmitters not only function within the nervous system but also play a significant role in tumor progression. In exploring the key genes involved in PNI progression, we identified that SDC3 and SEMA3D play significant roles. SDC3 serves as a common receptor for both MDK and the pleiotropic growth factor pleiotrophin (PTN), facilitating neural cell growth. Within the tumor microenvironment, necrotic tumor cells release extracellular PTN, which is then recognized by the SDC3 receptor, leading to neural proliferation (Alrawashdeh et al., 2019). However, some studies suggest that SDC3 upregulation in neural cells may cause PTN to accumulate around neurons, worsening nerve damage. Thus, PTN-SDC3 signaling could have dual roles in PNI development (Yao et al., 2013). In summary, the mechanism of tumor-associated PNI reflects complex interactions between tumor cells and the nervous system. This bidirectional relationship forms the basis for PNI formation and progression, though the precise molecular mechanisms remain intricate and warrant further study.

The aggrecan gene (AGRN) encodes a protein essential for neuromuscular junction development. This protein encodes several laminin G, Kazal-type serine protease inhibitor, and epidermal growth factor structural domains, with a core protein size of approximately 220 kDa, which is widely recognized for the role it plays at the neuromuscular junction (Williams et al., 2008). Researchers proposed that, in addition to its function at the neuromuscular junction, AGRN might also participate in the onset and progression of diverse tumors, including oral squamous cell carcinoma, hepatocellular carcinoma, and cholangiocarcinoma (Kawahara et al., 2014; Rivera et al., 2018; He et al., 2021; Somorácz et al., 2010; Yan et al., 2018). In oral squamous cell carcinoma, researchers found that silencing AGRN disrupts key cancer cell behaviors, including motility, proliferation, and invasion (Rivera et al., 2018). In cholangiocarcinoma, a primary liver cancer originating in bile duct cells, AGRN is also overexpressed, particularly in newly formed blood vessels. High AGRN expression is observed in the early stages of cholangiocarcinoma, whereas its expression decreases or is lost in later, poorly differentiated stages, suggesting that AGRN may play a vital role in supporting initial tumor growth (Iozzo et al., 2009). Additionally, another study showed that reducing AGRN protein levels through siRNA significantly decreases cell adhesion and migration, while enhancing tumor sensitivity to cisplatin treatment (Kawahara et al., 2014). These findings collectively highlight AGRN’s potential as a target for both early tumor progression and therapeutic intervention. However, the significance of AGRN in the development of colon cancer PNI has not been investigated. Considering the role of AGRN in the muscular synapses of the nervous system and its impact on tumor progression, combined with our identification of the factors that may influence the development of PNI in tumors in the previous article, it is reasonable to hypothesize that AGRN contributes to the progression of PNI. In this study, the relevant arguments were made using bioinformatics techniques and pathological immunohistochemical experiments.

## 2 Materials and methods

### 2.1 Bioinformatics dataset

In this study, the Cancer Genome Atlas (TCGA) and Ensembl databases were selected as the core datasets. We first extracted gene expression information, clinical case data, and immune infiltration data from the TCGA database. Subsequently, the gene set related to “perineural invasion” was extracted from the Ensembl dataset. Then, we cleaned and analyzed the data from these two datasets to obtain the core gene set for this study.

### 2.2 Data processing and analysis

In this study, R software was used for data cleaning and processing. First, the colon cancer data were downloaded from UCSC Xena and cleaned using the “tidyverse” package in R software. Duplicates, missing values, and other useless data were removed, and gene annotation was performed using Ensembl_ID. Subsequently, the “DESeq2″ package in the R software was used to screen the gene sets extracted from the TCGA database, with fold change (FC) > 1, log_2_FC > 0.4, and P < 0.05. To ensure a more comprehensive inclusion of candidate genes, we set a lower threshold. Current evidence suggests that this setting remains valid and reliable (Love et al., 2014; McDermaid et al., 2019). The truncation value is used to identify the set of genes that are highly expressed in tumor tissues. We searched for genes related to PNI using the keywords “neural proliferation,” “synapse formation,” and “neurotrophic factor” in Ensemble. The resulting gene sets were refined through survival analysis using the R package “survival” and “ggplot2.”

### 2.3 Bioinformatic exploration of the target genes

After identifying the target genes, we performed an extensive and in-depth exploration of the target genes using bioinformatics tools and existing large databases. The present study used clinical data obtained from TCGA to analyze the relationship between tumor staging and AGRN expression levels in colon cancer patients. The statistical method used was Kruskal–Wallis one-way ANOVA, and the statistical output and visualization were performed using R software. Simultaneously, we performed a pan-cancer analysis of the target gene using the TCGA and GTEx databases to investigate its expression levels in various gastrointestinal tumors. We used the R package “immunedeconv” to evaluate the correlation between AGRN and immune infiltration in various digestive tract tumors. Furthermore, methylation is a crucial factor influencing gene expression, and MethSurv is a web-based tool for survival studies that use DNA methylation data. We investigated the relationship between methylation levels of AGRN and prognosis using the MethSurv website (Modhukur et al., 2018).

### 2.4 Understanding gene function and associated pathways

Individual genes cannot perform their functions, and the performance of their functions depends on the gene pathways that are jointly composed of their upstream and downstream genes and their associated genes (Davidson and Levin, 2005). Hence, we explored the functional pathways that might be enriched in the set of target genes in Webgestalt (https://www.webgestalt.org/). An overexpression analysis was performed using a combination of KEGG and Reactome databases to identify the pathways of gene set enrichment (Liao et al., 2019).

Protein interaction networks (PPIs), which consist of links between proteins and their chaperones, play a pivotal role in various biological processes (Cheng et al., 2020). Gene expression data from TCGA were analyzed using Spearman correlation to assess the relationship between other genes and AGRN. We identified genes directly and indirectly associated with the target genes in the STRING. The interaction evidence was set as “experimental study, database, literature study, and co-expression”; the lowest interaction score was set as 0.400. The target gene-centered target gene network obtained from the STRING database was then analyzed using Cytoscape software (Franz et al., 2023). The ‘degree’ values of the genes were selected as the ranking criteria for the “proximity” between the associated genes and the core genes. Subsequently, the inter-gene relationships were mapped. Higher degree values indicate greater significance of the gene node in the network (García-García et al., 2015; Sciarra et al., 2018). Then, the gene enrichment pathway was mapped using the ClueGO + CluePedia plugin in Cytoscape (Bindea et al., 2009). We built a risk model to validate the gene sets’ prognostic significance. The feature selection process employed the Least Absolute Shrinkage and Selection Operator (LASSO) regression algorithm, utilizing a 10-fold cross-validation approach. Log-rank tests were used in KM survival analysis to compare survival differences between the two groups, and timeROC analysis was performed to evaluate the prediction model’s accuracy.

### 2.5 Validation of clinical data and pathological tissue samples

#### 2.5.1 Clinicopathological samples

Case data and tumor pathology specimens were collected from patients with primary colon cancer who underwent radical surgical resection at the First Hospital of Jilin University from 2013 to 2022. Patient data were gathered from pathology reports and case information, including age, gender, tumor grade, lymph node involvement, tumor markers (such as CA199, CA125, and CEA), tissue necrosis, MSI status, Ki67, P53 expression, and tumor outgrowth. Human specimens were obtained from the Department of Biobank, Division of Clinical Research, The first hospital of Jilin University.

#### 2.5.2 Sample inclusion and exclusion criteria

The patients were included for the data collection with the following criteria: All pathological samples were patients who were accurately diagnosed as primary colon cancer malignant tumors by pathologists after surgery; the clinical data were complete and available; the follow-up was accurate; no neoadjuvant radiotherapy, chemotherapy, or molecular targeted therapy was carried out; the patients’ basic vital signs were good, and they did not have a circulatory, neurological, endocrine system and other serious underlying diseases.

The patients were excluded from data collection due to the following criteria: Insufficient clinical data; inability to gather patient survival information during follow-up visits; patients with cardiovascular, neurological, or other serious underlying diseases; patients receiving neoadjuvant chemotherapy before and after surgery, molecular targeted therapy after surgery, and intraoperative and postoperative radiotherapy.

#### 2.5.3 Experimental methods

The gathered pathological specimens were fixed and sectioned, and then immunohistochemistry staining was performed to confirm the presence of AGRN expression in the tissue. Detailed detection and evaluation methods are described in the study by Cao et al. (2017). PNI was identified using S-100 staining (White et al., 2014). The expression levels of AGRN will be defined according to the following criteria: staining intensity is rated as 0,1,2,3 (no staining, weak, moderate, strong), while the proportion score is 1 or 2 (<30% of tumor cells or ≥30% of tumor cells). The total score, obtained by multiplying the intensity score and the proportion score, ranges from 0 to 6, with 0–2 classified as “negative” (low AGRN expression) and 3–6 as “positive” (high AGRN expression). Thank you again for your guidance.

#### 2.5.4 Statistical analysis of data

Statistical analysis was conducted using SPSS 26.0 software. Measurement data were expressed as mean ± standard deviation (X ± S), and intergroup comparisons were made using the rank sum test. Correlations were assessed using Spearman analysis, with statistical significance defined as *P* < 0.05.

## 3 Results

### 3.1 Bioinformatics results

#### 3.1.1 Acquisition of target genes

**FIGURE 1:**
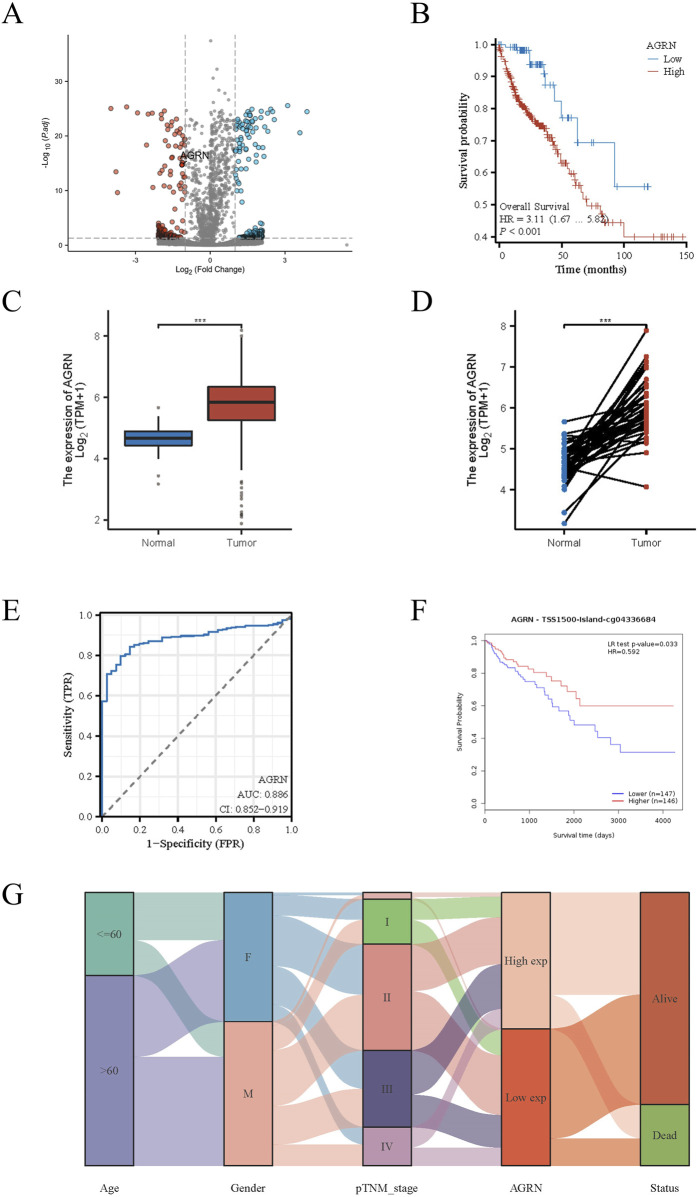
PNI-related genes in colon cancer. **(A)** Volcano plot displaying the expression levels of PNI-associated genes in colon cancer tissue. **(B)** AGRN Kaplan-Meier curve. **(C)** Comparison of AGRN expression between tumor and normal tissues. **(D)** Comparison of AGRN gene expression between tumor tissues and matched adjacent normal tissues. **(E)**. AUC Curve for Colon Cancer Prediction Based on AGRN Expression Levels. **(F)**. The relationship between AGRN-related methylation levels and patient prognosis. **(G)**. A mulberry plot depicting the association between AGRN and related clinical data.

A search of the Ensembl database yielded 1272 PNI-related genes. The above gene set was merged with the TCGA gene expression dataset, and 61 genes highly expressed in colon cancer were screened by differential analysis combined with clinical data (Figure 1A). The above 61 genes were further validated and screened against a large database, and the genes with low expression levels in colon cancer tissues and unrelated to prognosis were excluded from obtaining the target gene AGRN for this study. AGRN expression was correlated with poor prognosis (Figure 1B) and was notably higher in tumor tissues than in non-tumorous tissues (Figures 1C, D). Thus, AGRN was selected for more extensive research and exploration.

#### 3.1.2 Exploration and validation of AGRN in multiple databases

**FIGURE 2:**
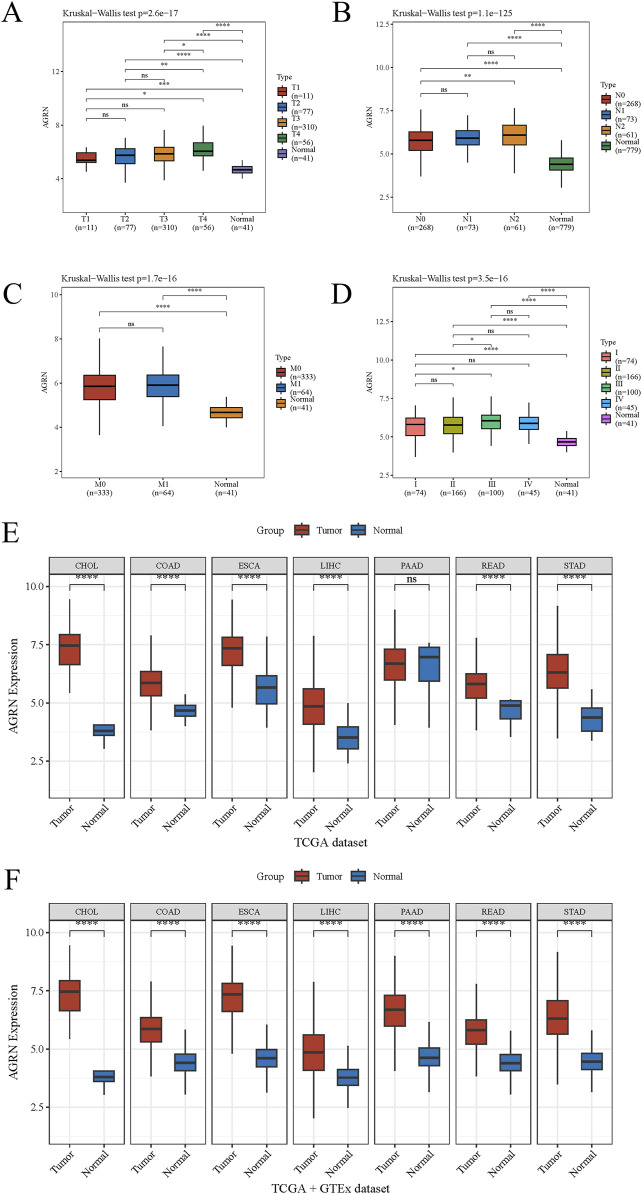
The association between AGRN and tumor staging and pan-cancer analysis. Tumor stage with AGRN: **(A)** T stage, **(B)** N stage, **(C)** M stage, and **(D)** TNM stage. **(E–F)** AGRN pan-cancer analysis.

**FIGURE 3:**
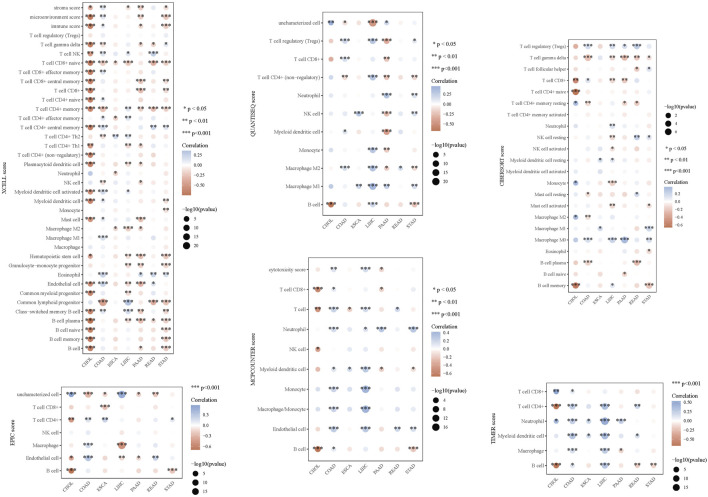
Immune infiltration analysis of AGRN in digestive system tumors using six different immune infiltration algorithms.

Leveraging clinical data from the TCGA database, we identified that elevated AGRN expression holds predictive significance for colon cancer development (Figure 1E). A Sankey diagram illustrates the relationship between AGRN expression levels and associated clinical data (Figure 1G). High methylation is commonly associated with decreased gene expression (Wu et al., 2024). We analyzed the correlation between AGRN methylation levels and prognosis, and the results revealed that the patients with higher expression of AGRN methylation had a better prognosis. This finding suggests that in these patients, the expression of the AGRN gene may be relatively low, and this low expression state is correlated with improved survival outcomes (Figure 1F). We found that AGRN expression levels tend to increase with higher tumor stages and greater lymph node metastases (Figures 2A–D). The pan-cancer analysis results showed that, in addition to high expression in colon cancer tissues, AGRN is also elevated in tumor tissues of cholangiocarcinoma, esophageal cancer, gastric cancer, and liver cancer compared to normal tissues (Figures 2E, F). The immune infiltration correlation analysis suggested that AGRN was positively correlated with most immune cells in cholangiocarcinoma, whereas it showed a negative correlation in colon cancer. The results varied slightly depending on the algorithm used (Figure 3).

#### 3.1.3 Functional analysis of AGRN and its associated genes and pathway exploration

**FIGURE 4:**
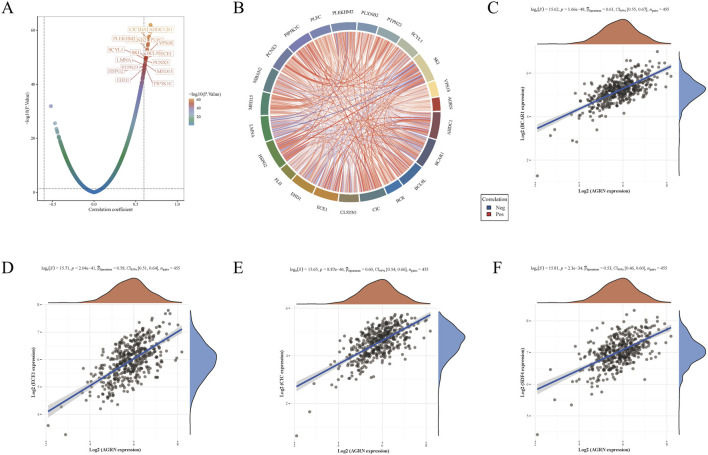
Gene expression correlation analysis. **(A)** A volcano plot depicting gene correlations centered on AGRN, with each point representing a gene and the color indicating the P-value. **(B)** Gene correlation heatmap is a detailed representation where blue lines denote negative correlations, and red lines indicate positive ones. **(C–F)** The correlation between AGRN and the expression levels of BCAR1, CIC, ECE1, and SDF4.

**FIGURE 5:**
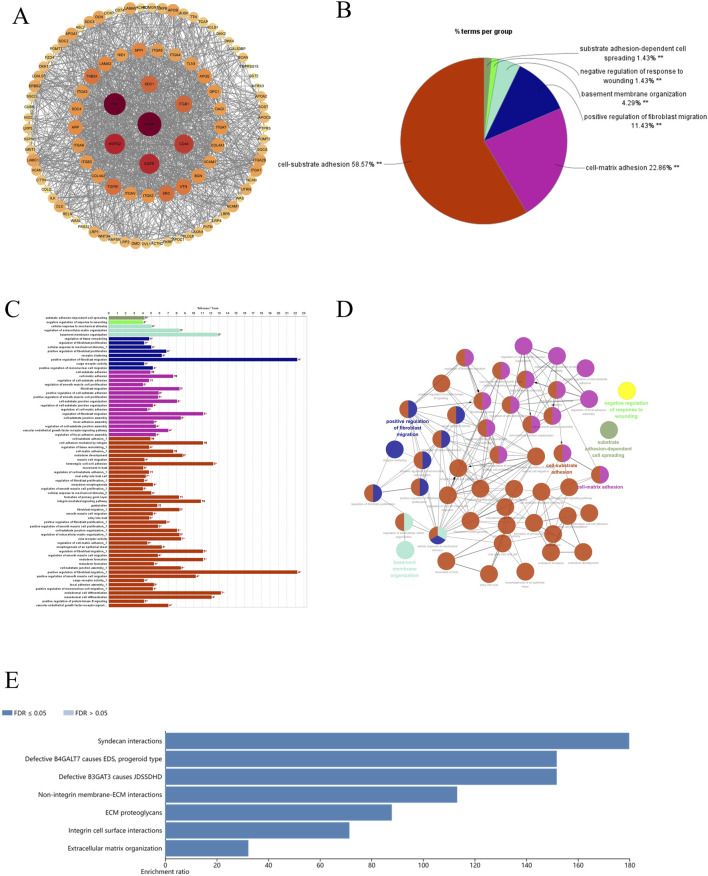
AGRN-associated genes and pathways display. **(A)** AGRN in the STRING database. **(B–D)** Pie and bar charts depicting pathways associated with AGRN-centered gene sets and their functional relationships. **(E)** Enrichment of genes with AGRN gene as the core gene in the Webgestalt database.

**FIGURE 6:**
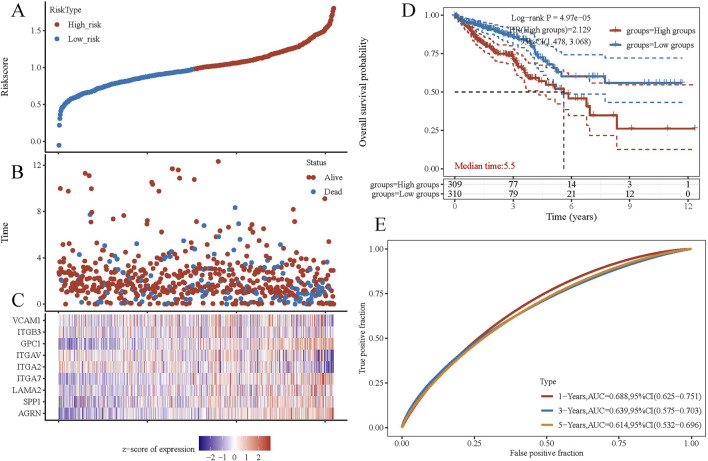
Gene set prognosis analysis. **(A)** Risk score curves. **(B)** Point plots of different survival states. **(C)** Heatmap of prognostic data for high-risk and low-risk groups. **(D)** KM curves. **(E)** ROC curves.

To explore the gene interaction network associated with AGRN, we first extracted the top genes positively correlated with AGRN (Figure 4A). We created a Circos plot to depict gene relationships (Figure 4B). BCAR1, CIC, ECE1, and SDF4 are significantly and positively correlated with AGRN expression (Figures 4C–F). We explored the interaction network centered on AGRN and mapped the relationship network between AGRN in string and its interacting genes (e.g., FN1, HSPG2, CD44, etc.) using Cytoscape (Figure 5A). Furthermore, we performed gene pathway enrichment analysis on these genes using the ClueGO plugin to find relevant pathways. These pathways can be classified into cell-substrate adhesion, cell migration, and cell basement membrane organization, closely related to tumor cell invasion. Cell-substrate adhesion accounted for 58.57%, and cell migration accounted for 11.43% (Figures 5B–D). Moreover, we performed a gene pathway enrichment analysis in Webgestalt at the same time. The gene set was significantly enriched in syndecan proteoglycan interaction, integrin-cell surface interaction, and extracellular matrix organization (Figure 5E). Then, we downscaled and constructed prognostic risk models for genes with degree values greater than 30 based on lasso regression (Figure 6A). Riskscore = (0.1318)*AGRN + (0.0321)*SPP1 + (0.0622)*LAMA2 + (0.1464)*ITGA7 + (−0.085)*ITGA2 + (−0.0143)*ITGAV + (0.1005)*GPC1 + (−0.2131)*ITGB3 + (−0.0122) *VCAM1. lambda. min = 0.0166 This model suggests that the above set of genes is an unfavorable factor for survival in colon cancer. Nine genes were in the risk model, with each gene having a weight, a negative number representing the gene as a protective gene, and a positive number representing the gene as a risk gene. The model AGRN identified SPP1, LAMA2, ITGA7, GPC1 as risk genes and ITGA2, ITGAV, ITGB3, VCAM1 as protective genes. According to the scatter plot, the number of points representing the dead patients gradually increases with the increased expression of the gene set in the model, whereas the number of points representing the surviving patients tends to decrease (Figures 6B, C). The KM plot shows that the prognostic survival of the group with high gene set expression is significantly lower than that of the group with low expression (Figure 6D). The AUC curve suggests that the gene set has a certain accuracy in predicting the development of colon cancer patients (Figure 6E).

### 3.2 Expression of AGRN in tumor tissue

#### 3.2.1 Relationship between AGRN and PNI in colon cancer

**FIGURE 7:**
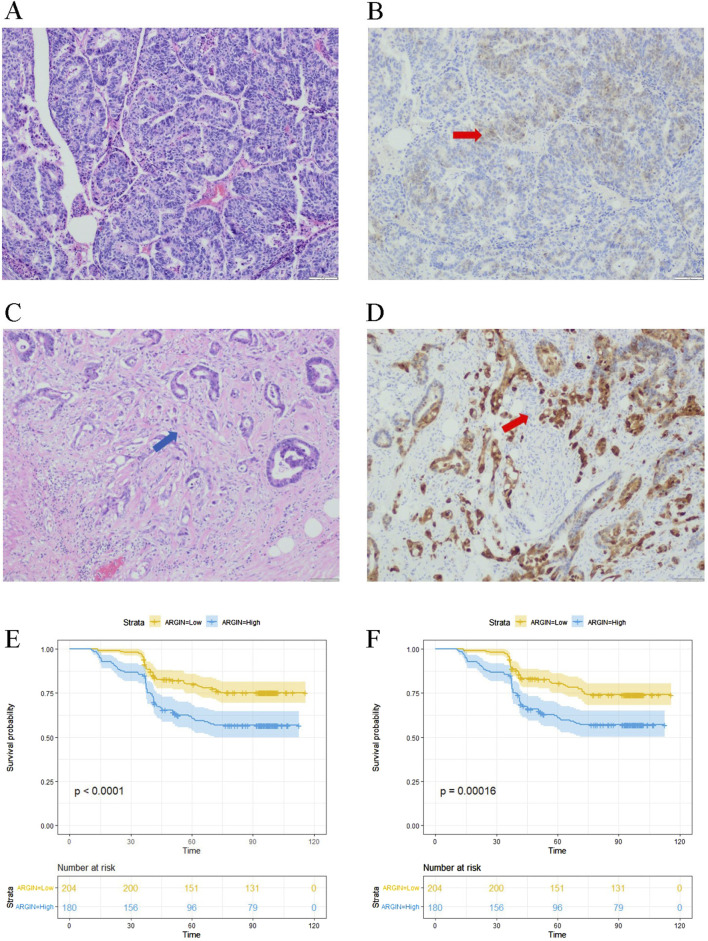
Immunohistochemical detection of AGRN. **(A)** PNI-negative tumor tissue. **(B)** AGRN immunohistochemical staining in PNI-negative cancer tissue, indicated by red arrows showing AGRN expression. **(C)** PNI-positive cancer tissue, with arrows highlighting nerve cells encircled by tumor cells. **(D)** AGRN immunohistochemical staining in PNI-positive cancer tissue, with arrows marking neural tissue. Seale bars = 50 μm. **(E–F)** KM curves showing overall OS **(E)** and DFS **(F)**.

The immunohistochemical results showed the expression of the AGRN gene in the tumor tissues of 384 colon cancer patients (Figures 7A–D). AGRN expression was more pronounced in PNI-positive colon cancer tissues compared to PNI-negative tissues (Table 1). A total of 384 colon cancer patient sections were counted for AGRN expression in this study. The results showed that AGRN was highly expressed in 95 (71.97%) of PNI-positive patients and low expression in 37 (28.03%). In the tumor tissues of PNI-negative patients, 85 (33.73%) patients showed high expression, whereas 167 cases showed low expression (66.27%). Our findings indicated that the incidence of PNI was significantly higher in patients with elevated AGRN expression compared to those with lower expression (*P* < 0.001), demonstrating a statistically significant difference.

**TABLE 1:** Association between AGRN expression levels, PNI status, and relevant clinical data.

Characteristics		AGRN		N	*p*
		Low expression	High expression		
PNI	Negative	85	167	252	0.000
	Positive	37	95	132	
Necrosis Stages	Negative	151	64	215	0.000
	Positive	53	116	169	
EGFR	Negative	80	51	131	0.025
	Positive	124	129	253	
CD34	Negative	125	111	236	0.937
	Positive	79	69	148	
CDX2	Negative	17	9	26	0.195
	Positive	187	171	358	
MSI	Stable	185	168	353	0.342
	Unstable	19	12	31	
TB	—	87.300 (75.7,98.4)	87.250 (74.6,99.0)	9.4 ± 6.4 (0–29)	0.896
Ki-67 status (%)	—	8.700 (4.5,13.8)	8.800 (4.0,13.4)	84.5 ± 13.8 (37.5–99.5)	0.854
Histology grade	Low-grade	181	158	339	0.773
	High-grade	23	22	45	
T stage	T1	5	3	8	0.035
	T2	30	11	41	
	T3	135	126	261	
	T4	34	40	74	
N stage	N0	96	42	138	0.000
	N1	62	75	137	
	N2	46	63	109	
TNM stage	Ⅰ	33	10	43	0.000
	Ⅱ	63	32	95	
	Ⅲ	108	138	246	
LVI	Absent	111	72	183	0.005
	Present	93	108	201	
CEA	≤5 ng/mL	29	10	39	0.005
	>5 ng/mL	175	170	345	
CA125	≤35 U/mL	36	13	49	0.002
	>35 U/mL	168	167	335	
CA199	≤37 U/mL	20	5	25	0.005
	>37 U/mL	184	175	359	

#### 3.2.2 The association between AGRN and clinicopathological data

We analyzed clinical data from 384 patients, including gender, age, tumor histological stage, lymph node metastasis, and tumor markers, by reviewing their clinical case records (Table 1). We statistically analyzed the expression of AGRN with various clinical and pathological data of the patients. The results showed that the expression of AGRN was not significantly different between colon and rectal cancer patients. However, statistically significant differences were observed in tumor T stage, lymph node metastasis, tumor necrosis, EGFR, and tumor markers, and in general, the higher expression of AGRN was more pronounced in patients with later stage and lymph node metastasis. No notable difference was observed in AGRN expression levels with respect to immunohistochemical markers, including CD34, CDX2, and MSI. We obtained patient survival curves by tracking the survival of 384 patients and combining the patients’ AGRN expression levels. The findings indicated that those with elevated AGRN expression had reduced levels of disease-free survival and overall survival compared to those with reduced AGRN expression (Figures 7E, F).

### 3.3 Relationship between clinicopathological data and the occurrence of PNI

We explored the relationship between PNI occurrence and various clinical factors. (Table 2). The occurrence of PNI in colon cancer patients was not significantly related to gender but had a certain correlation with age, and the chance of PNI in colon cancer patients would be higher with increasing age. However, no obvious relationship was found between the occurrence of PNI and tumor markers. The occurrence of PNI had a significant correlation with the TNM stage of the tumor, and the probability of PNI occurrence was considerably higher in patients with later-stage tumors. Patients diagnosed with later-stage tumors had a significantly higher probability of developing PNI. Patients with lymph node metastases were also more likely to have PNI in their tumors, and the difference was statistically significant. We further explored the association between tumor necrosis, microsatellite instability (MSI), Ki67, tumor outgrowth, and PNI in colon cancer. The Ki67 is a crucial indicator for determining if cells are in the division cycle; a positive value of Ki67 suggests that tumor cells are in an active state and grow faster (Liu et al., 2023). Tumor outgrowth also tends to represent more active tumor invasion and is an independent prognostic marker for colon tumors (Cho and Kakar, 2018). The present results showed that Ki67, tumor outgrowth, EGFR, and necrotic status were significantly correlated with PNI in colon cancer, with *p* < 0.001, whereas tumor MSI, CD34, and CDX2 were not significantly associated with the occurrence of PNI in patients.

**TABLE 2:** The relationship between PNI status and patient clinical information as well as pathological data.

Characteristics		PNI status		N	*P*
		Negative	Positive		
Gender	Female	155	77	234	0.449
	Male	95	55	150	
Age	—	63.9 ± 12.1 [26–89]	132	67.1 ± 11.3 [24–93]	0.013
CEA	≤5 ng/mL	25	14	39	0.833
	>5 ng/mL	227	118	345	
CA125	≤35 U/mL	31	18	49	0.710
	>35 U/mL	221	114	335	
CA199	≤37 U/mL	16	9	25	0.860
	>37 U/mL	236	123	359	
Histology grade	Low-grade	231	108	339	0.004
	High-grade	21	24	45	
T stage	T1	8	0	8	<0.001
	T2	41	0	41	
	T3	162	99	261	
	T4	41	33	74	
N stage	N0	135	3	138	<0.001
	N1	64	73	137	
	N2	53	56	109	
TNM stage	Ⅰ	43	0	43	<0.001
	Ⅱ	92	3	95	
	Ⅲ	117	129	246	
LVI	Absent	156	27	183	<0.001
	Present	96	105	201	
Necrosis Stages	Negative	173	42	215	<0.001
	Positive	79	90	169	
MSI	Stable	232	121	353	0.892
	Unstable	20	11	31	
P53	Wild-type	82	47	129	0.546
	Mutant	170	85	255	
TB	—	8.4 ± 5.8 [0–21]	11.2 ± 6.9 [0–29]	9.4 ± 6.4 [0–29]	<0.001
Ki-67 status (%)	—	83.0 ± 14.2 [37.5–99.5]	87.4 ± 12.4 [52.5–99.5]	84.5 ± 13.8 [37.5–99.5]	0.003
EGFR	Negative	34	131	97	0.012
	Positive	98	253	155	
CD34	Negative	88	236	148	0.129
	Positive	44	148	104	
CDX2	Negative	7 (5.30)	26	19 (7.54)	0.407
	Positive	125 (94.70)	358	233 (92.46)	

## 4 Discussion

Bioinformatics analysis showed that AGRN was overexpressed in colon tumor tissues and adversely affected patient survival. Considering the significance of AGRN, we analyzed genes closely associated with it and explored their potential interactions. AGRN is strongly and favorably associated with several genes, which have a role in the proliferation and invasion of cancer cells. BCAR1 showed a positive correlation with AGRN. Previous studies have indicated that BCAR1 can interact with TP53 and FLOT1, thereby enhancing the invasive ability of cancer cells (Guo A. K. et al., 2021; Wang R. et al., 2023). ECE1 activates multiple pathways related to cell proliferation, survival, and invasion, as demonstrated in the studies associated with colon cancer stem cells (Chi et al., 2021). In addition to the invasive capacity of cancer cells, changes in the tumor microenvironment also play a role in the process of cancer metastasis. Cancer-associated fibroblasts (CAFs) are crucial in promoting cancer progression (Chen and Song, 2019; Chen et al., 2021). Stromal cell-derived factor 4 (SDF4) responds to anticancer drugs by activating CEBPD within CAFs, establishing a favorable environment for angiogenesis in tumor cells and facilitating distant metastasis. Furthermore, SDF4 binds to CXCR4, leading to the upregulation of VEGFD by stimulating the ERK1/2 and p38 pathways in endothelial cells (Chi et al., 2021). Apart from its role in tumor cell invasion, Capicua (CIC), a member of the High Mobility Group-box (HMG-box) transcriptional repressor superfamily, is significantly correlated with its target gene AGRN (Hwang et al., 2020). Researchers indicated that CIC knockout leads to abnormal neurodevelopment *in vivo* and defects in the differentiation and proliferation of neural stem cells *in vitro*, highlighting the critical role of CIC in neural cell proliferation (Yang et al., 2017). Based on these findings, we infer that AGRN and its associated genes drive the proliferation and invasiveness of tumor cells and promote neural cell proliferation. Collectively, these effects may contribute to tumor PNI.

Subsequently, we further explored the associated genes in the STRING database with AGRN as the core and performed gene function enrichment analysis on them. Based on the information in the STRING database, we constructed a gene network diagram using the inter-gene degree as a benchmark. From this diagram, we selected the genes at the core of the network diagram, such as FN1, Hspg2, and Src, for further exploration. The above genes were found to play different roles in the tumor stroma, angiogenesis, tumor cell proliferation, migration, and nervous system development (Zhao et al., 2022; Zhang et al., 2022; Ma et al., 2018; Kalia et al., 2004; Dehm and Bonham, 2004). Beyond its role in tumor cells, Src and other Src family kinases are widely expressed and prevalent in neurons within the central nervous system (CNS). Src has been associated with proliferation and differentiation during CNS development (Kalia et al., 2004). As emphasized in the present study, gene enrichment analysis suggests that AGRN and its related pathways may be involved in cell-matrix adhesion and cell migration. Immune infiltration analysis revealed that the target gene expression level in colon cancer is negatively associated with T cells, endothelial cells, and macrophages, confirmed by several immune infiltration algorithms. Based on this negative correlation, we speculate that high expression of this gene may inhibit the relevant cells into the tumor site, thereby affecting the tumor’s immune escape mechanism (Me et al., 2019; Li et al., 2016; Kao et al., 2022). Overall, the AGRN-centered gene set significantly influences tumor cell adhesion and invasion, tumor angiogenesis, and neurodevelopment.

Apart from bioinformatics mining, this study further explored the expression of AGRN in colon cancer and the possible influencing factors of PNI occurrence and development by immunohistochemical experimental studies. Our experimental results confirmed that AGRN showed elevated expression in PNI-positive tumor tissues and reduced patient and disease-free survival, thereby confirming the results obtained from the bioinformatics analysis. The AGRN gene was found to be an unfavorable factor for the survival of colon cancer patients and was strongly associated with the PNI of the tumors. Further statistical analysis of patients’ clinicopathological data revealed that high AGRN expression was positively associated with tumor stage and lymph node metastasis, suggesting that AGRN acts as a promoter of colon cancer progression. AGRN was correlated with epidermal growth factor receptor (EGFR) protein and tumor necrosis status in immunohistochemical results. Existing studies have shown that EGFR, a transmembrane protein, is closely associated with angiogenesis in the tumor region and tumor invasion, emerging as a crucial indicator for guiding targeted therapies (Kasi et al., 2023). In the tumor microenvironment, prolonged inflammation, stress, and hypoxia lead to tumor necrosis, and the release of necrosis factors promotes tumor progression and invasion (Karsch-Bluman et al., 2019). The association between AGRN, EGFR, and tumor necrosis further reinforces the role of AGRN in tumor progression and offers valuable insights for targeted cancer therapy. The result is consistent with our bioinformatics analysis, suggesting that AGRN and its related genes are involved in tumor development, progression, and PNI.

Furthermore, we investigated the relationship between the occurrence of PNI and other pathological indicators using case data from our institution. Our findings suggest that enhanced tumor cell proliferation, invasiveness, and outgrowth likely drive the occurrence of PNI. Moreover, this study does present limitations. First, this study presents results from a single center, and future research could strengthen validation by increasing sample size and including data from multiple centers. Second, this study primarily uses immunohistochemistry and statistical methods to examine AGRN’s role in PNI, without fully investigating the molecular mechanisms by which AGRN may mediate PNI. Future research should incorporate molecular biology techniques, such as *in vitro* cell assays and *in vivo* animal models, to further elucidate how AGRN facilitates PNI progression. Lastly, while this study provides valuable insights into the relationship between PNI and clinicopathological indicators, correlation analyses cannot establish causality. Prospective studies and clinical trials will be crucial to confirm these findings and assess AGRN’s potential as a biomarker and therapeutic target for PNI.

## 5 Conclusion

To summarize, this study explored the expression and clinical relevance of the PNI-related gene AGRN in tissue samples using bioinformatics, immunohistochemistry, and statistical analyses to identify factors influencing PNI. This research provides a foundation for understanding PNI mechanisms in tumors. Our findings showed that AGRN is overexpressed in colon cancer and is associated with the development of PNI, T stage, lymph node metastasis, and proliferation index. Thus, AGRN may promote colon cancer proliferation and PNI development, indicating its potential as both therapeutic and diagnostic markers for colon cancer.

## Data Availability

The datasets presented in this study can be found in online repositories. The names of the repository/repositories and accession number(s) can be found in the article/supplementary material.

## Abbreviations

PNI, Perineural invasion; AGRN, aggrecan gene; TCGA, The cancer genome atlas; PPIs, Protein interaction networks; NGF, nerve growth factor; PTN, pleiotrophin; MDK, midkine; LASSO, Least absolute shrinkage and selection operator; KM, Kaplan-Meier; MSI, Microsatellite instability; DFS, Disease-free survival; OS, Overall survival; FC, Fold change; DEG, Differentially expressed genes; H&E, Hematoxylin-eosin; CAFs, Cancer-associated fibroblasts; SDF4, Stromal cell-derived factor 4; CIC, Capicua; CNS, central nervous system; EGFR, epidermal growth factor receptor.
